# Elevated neutrophil extracellular traps formation driven by the HPV-mediated CXCL8-CXCR1/2-ROS-p21 axis promotes the growth and metastasis of cervical cancer

**DOI:** 10.3389/fimmu.2026.1861481

**Published:** 2026-08-20

**Authors:** Xin Wang, Alimire Julaiti, Aikelimu Aisikaer, Hongjian Wei, Runda Jie, Xinyan Liu, Yonghua Shi

**Affiliations:** 1 Department of Pathology, Basic Medical College, Xinjiang Medical University, Urumqi, Xinjiang, China; 2 Department of Pathology, Urumqi Friendship Hospital, Urumqi, Xinjiang, China; 3 Clinical Medicine Department, Xinjiang Medical University, Urumqi, Xinjiang, China; 4 Xinjiang Key Laboratory of Molecular Biology for Endemic Diseases, Urumqi, Xinjiang, China

**Keywords:** neutrophil extracellular traps, human papillomavirus, CXCL8, metastasis, cervical cancer

## Abstract

**Background:**

The role of neutrophil extracellular traps (NETs) in cervical cancer (CC) progression, particularly their association with human papillomavirus (HPV) infection, remains unclear.

**Methods:**

We analyzed public single-cell RNA sequencing data to explore neutrophil heterogeneity in the HPV-associated CC microenvironment and quantitatively assessed NETs in clinical tissues and serum from normal subjects, HPV-negative, and HPV-positive CC patients. *In vitro*, CC cell lines and normal epithelial cells were treated with neutrophil-conditioned medium, followed by phenotypic evaluation using EdU, Transwell, and vasculogenic formation assays. *In vivo* validation was conducted using subcutaneous xenograft and tail-vein metastasis models. Potential mechanisms of HPV-regulated NETs formation were further investigated through co-culture, immunoblotting, and ROS detection.

**Results:**

Single-cell transcriptomic analysis revealed a characteristic neutrophil subpopulation (N4) in HPV-positive tumor microenvironments, whose gene expression signature is highly correlated with NETs formation capability. NETs were elevated in CC tissues and sera, especially in HPV-positive tumors. High tissue levels of the NETs-specific marker Cit-H3 correlated with larger tumor diameter, deep stromal invasion and poor differentiation. Functionally, NETs promoted proliferation, angiogenesis, invasion and migration of CC cells; *in vivo*, experiments showed NETs drove tumor growth and pulmonary metastasis, effects abolished by DNase 1-mediated NETs degradation. Mechanistically, HPV-positive cancer cells secrete substantial amounts of CXCL8, which activates CXCR1/2 receptors, thereby inducing reactive oxygen species production and p21 upregulation to drive neutrophil extracellular trap formation. Targeting this axis reversed NETs generation.

**Conclusion:**

The HPV-triggered CXCL8-CXCR1/2-ROS-p21 pathway drives NETs formation that accelerates CC growth and metastasis. Circulating NETs represent a potential prognostic biomarker.

## 1 Introduction

Cervical cancer (CC) ranks as the fourth leading cause of cancer-related deaths among women worldwide and represents the second most common cause of cancer mortality in young women aged 20~39 (1). Persistent infection with human papillomavirus (HPV) has been established as a major causative factor in cervical carcinogenesis (2). However, the virus alone is inadequate to fully account for the entire process of malignant transformation (3). It is currently believed that the remodeling of the microenvironment (TME) triggered by HPV infection serves as a key driver of CC progression (4, 5). Nevertheless, the specific molecular and cellular events underlying this process remain to be systematically elucidated.

During persistent chronic infection caused by HPV, a chronic inflammatory microenvironment develops in the local tissue. Neutrophils, which serve as the first line of the human immune defense, are extensively recruited into the tumor microenvironment (6). These accumulated tumor-associated neutrophils (TANs) within the local tumor tissue exhibit high plasticity and, under the regulation of complex microenvironmental signals, can exert powerful modulatory effects on tumor progression (7, 8). Of note, neutrophil extracellular traps (NETs) released by TANs have attracted growing attention as a distinctive form of immune response. NETs are intricate network structures composed of decondensed chromatin embedded with various antimicrobial proteins, such as citrullinated histone H3 (Cit-H3), matrix metalloproteinase-9 (MMP9) and myeloperoxidase (MPO) (9). Initially recognized as a pathogen defense mechanism, growing evidence has revealed their roles in tumorigenesis, metastasis, and immune evasion (10, 11). In CC, NETs have been shown to potentiate metastasis through the TLR2/P38-MAPK/ERK/NF-κB signaling axis (12), indicating that they function not merely as inflammatory by-products but as active accelerators of malignant progression.

CXC chemokines are a class of cytokines that mediate neutrophil chemotaxis and trafficking through their specific, high-affinity binding to CXC chemokine receptors (CXCRs) (13). CXCL8, a member of the ELR^+^ CXC subfamily, is notably the most potent and abundantly produced factor for neutrophil activation (14). Previous studies have shown a positive correlation between NETs density and CXCL8 protein expression (15, 16). However, it remains unclear whether CXCL8 contributes to the development and progression of cancer, especially HPV-related CC by specifically inducing NETs formation.

In this study, we utilized clinical samples of HPV-associated CC, combined with *in vitro* and *in vivo* model systems, to investigate the role of HPV-induced NETs in driving CC invasion and metastasis, as well as the underlying molecular mechanisms.

## 2 Materials and methods

### 2.1 Clinical sample collection

Fresh tissue specimens were obtained from the Biobank of the Affiliated Tumor Hospital of Xinjiang Medical University (April 2017~January 2022). The study enrolled 39 HPV-positive CC patients aged 35~74 years. Among the malignant lesions, 20 cases were FIGO stage I and 19 were stage II–III; no lymph-node metastasis occurred in 29 and nodal involvement in 10; superficial and deep cervical stromal invasion were present in 23 and 16 cases, respectively; 23 were moderately differentiated and 16 poorly differentiated. Histologically, 35 were squamous-cell carcinomas, 3 were adenocarcinomas, and 1 was adenosquamous carcinoma. An additional 50 formalin-fixed paraffin-embedded samples (10 normal cervices, 10 HPV-negative cancers, 30 HPV-positive cancers) and 20 serum samples (6 healthy controls, 4 HPV-negative cancer patients, 10 HPV-positive cancer ones) were provided by the same institution. The study protocol was approved by the Ethics Committee of the Affiliated Tumor Hospital of Xinjiang Medical University (Approval No. 2023BC030), and written informed consent was obtained from all participants.

### 2.2 Cell lines

The human HPV-positive CC cell lines HeLa (CVCL_0030) and SiHa (CVCL_0032), the human HPV-negative CC cell line C-33A (CVCL_1094), the human normal cervical epithelial cell line (HCerEpiC), and the human umbilical vein endothelial cell line (HUVEC) were obtained from the Pathology Laboratory of Xinjiang Medical University. The HL-60(#CL-0110) (CVCL_0002) cell line was purchased from Wuhan Pricella Biotechnology Co., Ltd. The mouse CC cell line U14 (#ZQ0948) (CVCL_9U56) was obtained from the Shanghai Zhong Qiao Xin Zhou Biotechnology Co., Ltd. DMEM (Gibco, USA) was used to cultivate HeLa, SiHa, C-33A, HCerEpiC, HUVEC and U14 cell lines, and IMDM medium (Pricella, China) was used to cultivate HL-60 cell line. All cell lines were cultured at 37 °C in a humidified incubator with 5% CO_2_, and only mycoplasma-free cells were used in all experiments. DMEM (Gibco, USA) was used to cultivate HeLa, SiHa, C-33A, HCerEpiC, HUVEC and U14 cell lines, which was supplemented with 10% heat-inactivated fetal bovine serum (FBS, Gibco, USA), 100 U/mL penicillin, and 100 μg/mL streptomycin (Gibco, USA), and HL-60 cell line were cultured in IMDM medium (Pricella, China), with 20% heat-inactivated fetal bovine serum (FBS, Gibco, USA), 100 U/mL penicillin, and 100 μg/mL streptomycin (Gibco, USA), at 37 °C in a humidified incubator with 5% CO_2_, and only mycoplasma-free cells were used in all experiments.

### 2.3 Bioinformatics analysis

#### 2.3.1 Data acquisition

The CC datasets GSE151666, GSE197461 and GSE208653 were acquired from the NCBI Gene Expression Omnibus (GEO) database (https://www.ncbi.nlm.nih.gov/geo/). NETs-related genes were retrieved from the GeneCards database (https://www.genecards.org/) by filtering for protein-coding genes with a relevance score > 10.

#### 2.3.2 Differential-expression analysis

The cervical-cancer RNA-sequencing dataset GSE151666 was imported into RStudio (v4.4.1). Ensembl IDs in the expression matrix were converted to gene symbols, and the data were normalized. Genes with expression < 1 across all samples were filtered out. Samples were classified as HPV-negative or HPV-positive based on the provided metadata, and expression values were log_2_-transformed. Differentially expressed genes were identified with the limma package using adjusted *P* < 0.05 and an absolute fold change > 1.2 as cut-offs.

#### 2.3.3 Single-cell transcriptomic analysis

Two HPV-negative cervical-cancer samples from dataset GSE197461 and three HPV-positive samples from dataset GSE208653 were selected. The Harmony algorithm was implemented to mitigate batch effects across distinct datasets. Clusters were generated at a resolution of 0.5 and annotated into major cell types and neutrophil subsets; cell-cycle status inferred by PCA did not influence clustering. Non-linear dimensionality reduction and visualization were performed with UMAP and t-SNE. Neutrophil clusters from HPV-positive tumors were subjected to functional enrichment to identify NETs-associated subpopulations. Trajectory inference and pseudotime analysis were conducted using Monocle 3 to uncover driver genes governing NETs subpopulation formation, and Louvain community detection was applied to define characteristic gene modules of the NETs subset.

#### 2.3.4 Selection of NETs-related core genes

To identify NETs-associated core genes within the HPV-positive tumor microenvironment, we integrated three gene sets and identified their intersection: NETs-related protein-coding genes with a relevance score >10 retrieved from GeneCards; differentially expressed genes between HPV-positive and HPV-negative tumors derived from bulk transcriptome analysis; and the NETs-related neutrophil gene module (Module 5) identified by single-cell analysis. Genes common to all three sets were defined as core NETs-related genes and used for downstream investigations.

#### 2.3.5 Survival, immune infiltration, and drug sensitivity analyses

Transcriptomic and clinical data of cervical cancer patients were obtained from the TCGA-CESC dataset. RNA-sequencing data and corresponding survival information were processed using R software with the survival and survminer packages. Among 306 tumor samples, 292 cases with complete survival records (follow-up period: 0–18.7 years) were included in the survival analysis. Patients were stratified into high- and low-CXCL8 expression groups based on the median expression level, and overall survival (OS) was compared using Kaplan–Meier curves with the log-rank test. Immune infiltration analysis was performed using the CIBERSORT algorithm to estimate the relative abundance of 22 immune cell subtypes. Based on the immune infiltration results, Spearman’s correlation analysis was further conducted to evaluate the correlation between CXCL8 expression and the abundance of differentially infiltrated immune cells. Drug sensitivity data were obtained from the Genomics of Drug Sensitivity in Cancer (GDSC) database (https://www.cancerrxgene.org/). The “oncoPredict” R package was used to assess the association between CXCL8 expression levels and drug sensitivity, as measured by the half-maximal inhibitory concentration (IC_5__0_).

### 2.4 HL-60 induction

Notably, primary human neutrophils exhibit limited yield, short ex vivo lifespan, and prominent donor-to-donor heterogeneity, all of which complicate repeated and reproducible functional assays. To address this issue and ensure experimental consistency and reproducibility, we drew on the published evidence that HL-60 cells differentiated with 1 μmol/L all-trans retinoic acid (ATRA, #R2625, Sigma-Aldrich, USA) for 5 days develop morphological and functional characteristics resembling mature neutrophils, making them a well-established surrogate model to dissect the molecular mechanisms underlying NET formation. Previous studies have demonstrated that approximately 80% of HL-60 cells differentiate into neutrophil-like cells upon ATRA or DMSO induction, accompanied by a significant inhibitory effect on the proliferation of the remaining undifferentiated HL-60 cells (17–19). To induce HL-60 cells to differentiate into neutrophil-like cells, 3×10^5^ HL-60 cells were seeded per milliliter and cultured for 5 to 6 days in IMDM medium containing 1 mol/L ATRA. HL-60 differentiation was assessed by Giemsa staining (#C0131, Beyotime, China) and the resulting neutrophil-like cells are hereafter referred to as neutrophils.

### 2.5 Preparation of conditioned medium

Neutrophils (1×10^6^ cells/mL) were cultured in serum-free IMDM. For NETs induction, cells were stimulated with PMA (25 nmol/L, #HY-18739, MedChemExpress, USA) for 4 hours. For NETs degradation control, cells were treated with PMA together with DNase 1 (100 U/mL, #HY-108882, MedChemExpress, USA) for 4 hours. For PAD4 inhibition, cells were pre-incubated with GSK484 (10 μmol/L, #HY-100514, MedChemExpress, USA) for 30 minutes prior to PMA stimulation for 4 hours. NETs and adherent neutrophils were then harvested and centrifuged for 10 minutes at 450 × g, 4 °C, to obtain cell-free, NETs-rich supernatants designated as conditioned medium (CM), which was aliquoted and stored at -80 °C for later use.

### 2.6 5-Ethynyl-20-deoxyuridine assay

HCerEpiC, C-33A, SiHa and HeLa cells treated with CM (Neu), CM (Neu + PMA) or CM (Neu + PMA + DNase 1) were seeded into 24-well plates at 3 × 10^4^ cells per well and cultured for 24 hours at 37 °C, 5% CO_2_. Cell proliferation was assessed with the EdU Cell Proliferation Kit (#C0075, Beyotime, China). Images were captured using an inverted fluorescence microscope (Leica, Germany).

### 2.7 Matrigel tube formation assay

Matrigel (50 μL/well; #356234, Corning, USA) was added to each well of a 96-well plate and incubated at 37 °C for 30 min to solidify. To obtain CM for tube formation assays, HCerEpiC, C-33A, SiHa, and HeLa cells were exposed to the indicated NETs-containing CM (from Neu, Neu+PMA, or Neu+PMA+DNase 1 groups) for 24 hours. The supernatants were then collected, centrifuged (450 × g, 10 min, 4 °C), and used to treat HUVECs. HUVECs were seeded onto the coated plates at a density of 3 × 10^4^ cells per well and cultured with the collected CM. HUVECs were then seeded onto the coated plates at a density of 3 × 10^4^ cells per well and treated with the indicated CM. After 6 hours of incubation, capillary-like structures were visualized and quantified under an inverted light microscope.

### 2.8 Cell invasion and migration assay

Transwell assays were performed using 24-well plates. For the cell invasion assay, the inserts (8 μm pore size; Corning, USA) were pre-coated with Matrigel (60 μL/well; #356234, Corning, USA). HCerEpiC, C-33A, SiHa, and HeLa cells (5 × 10^4^ cells/well) were resuspended in 200 μL of serum-free medium and seeded into the upper chamber. The lower chamber of the 24-well plate was filled with various types of CM containing 20% FBS. After 48 hours of incubation, the cells were fixed with 4% paraformaldehyde for 30 min and stained with 0.1% crystal violet for 20 minutes. The inserts were then gently washed with PBS, and non-invading cells on the upper surface of the membrane were carefully removed using a cotton swab. The invaded cells were visualized and counted under an inverted microscope. For the cell migration assay, a similar procedure was followed. Briefly, HCerEpiC, C-33A, SiHa, and HeLa cells (5 × 10^4^ cells/well) were seeded into uncoated Transwell inserts (8 μm pore size). All subsequent steps were performed identically to the invasion assay protocol as described above.

### 2.9 Western blotting

Cells were lysed in RIPA buffer containing 1% protease inhibitor. Protein concentration was determined using a BCA assay kit. Equal amounts of protein extracts were heated at 100 °C for 10 minutes, separated by SDS-PAGE, and transferred onto polyvinylidene fluoride (PVDF, #3010040001, Sigma-Aldrich, USA) membranes. The membranes were then blocked with 5% skim milk for 2 hours at room temperature and incubated overnight at 4 °C with the following primary antibodies: anti-Cit-H3 (#ab281584, Abcam, UK), anti-MPO (#66177-1-Ig, Proteintech, China), anti-MMP9 (#10375-2-AP, Proteintech, China), anti-E-cadherin (#AF0131, Affinity, China), anti-Vimentin (#AF7013, Affinity, China), anti-VEGF (#AF5131, Affinity, China), anti-PCNA (#AF0239, Affinity, China), anti-CXCL8 (#DF6998, Affinity, China), and anti-β-actin (#66009-1-Ig, Proteintech, China), anti-p21(#R381102, zenbio, China). CXCR1/2 inhibitor Reparixin (#HY-15251, MedChemExpress, USA), NADPH oxidase inhibitor diphenyleneiodonium chloride (DPI, #HY-100965, MedChemExpress, USA), p21 inhibitor UC2288 (#HY-112780, MedChemExpress, USA). Overnight, the membranes were washed five times with TBST and incubated with horseradish peroxidase (HRP)-conjugated secondary antibodies for 1 hour at room temperature. After five additional washes with TBST, the blots were developed using the Western Bright ECL detection system (Bio-Rad, USA). Each experiment was performed with three biologically independent samples. Protein expression levels were quantified using ImageJ software.

For clinicopathological correlation analysis, Cit-H3 and MMP9 protein expression levels were quantified from Western blot bands in 39 HPV-positive CC tissues. Since median-based grouping did not yield statistically significant differences, the mean value of protein expression was used as the cut-off to divide patients into high- and low-expression groups. The associations between Cit-H3/MMP9 expression levels and clinicopathological parameters were then analyzed using the χ² test or Fisher’s exact test, as appropriate. For univariate analysis of 5-year survival, the chi-square test (or Fisher’s exact test when appropriate) was used to compare survival rates between groups. Multivariate analysis was performed using the Cox proportional hazards regression model to identify independent prognostic factors. Variables with a P value < 0.05 in the univariate analysis were entered into the multivariate model.

### 2.10 *In vivo* tumor growth and metastasis

Subcutaneous xenograft and tail vein metastasis models were established using 5~6-week-old female C57BL/6 mice obtained from the Laboratory Animal Center of Xinjiang Medical University. Subcutaneous injection: A total of 18 C57BL/6 mice were randomly assigned to three groups (n=6 per group). U14 cells were co-cultured for 24 hours with neutrophils (Neu) at a 1:1 ratio, which were either left untreated or treated with PMA and DNase 1. After 24 hours of co-culture, neutrophils (Neu) underwent apoptosis and released NETs. U14 cells were then resuspended in 200 μL of cold PBS. The three experimental groups were designated as follows: Neu + U14, Neu (PMA) + U14, and Neu (PMA + DNase 1) + U14. A suspension of cells (2 × 10^6^ cells per mouse) from each group was injected subcutaneously into the right flank. Tumor growth was monitored every two days using digital calipers. Tumor volume was calculated using the formula: Volume = L × W²/2, where L represents the length and W the width of the tumor. Tumor growth curves over time were analyzed using two-way ANOVA. Tail vein injection: Another 18 C57BL/6 mice were randomly divided into three groups (n=6 per group). The three experimental groups were designated as follows: U14, LPS + U14, and LPS + U14 + DNase 1. Systemic inflammation was induced by intraperitoneal (i.p.) injection of lipopolysaccharide (LPS, 10 μg per mouse, #L2880, Sigma, USA) or PBS. Six hours later, U14 cells (1×10^6^ cells per mouse) were injected via the tail vein, followed by daily i.p. administration of DNase 1 (10 U per mouse, #HY-108882, MedChemExpress, USA) or PBS. On day 30, mice were anesthetized with an i.p. injection of sodium pentobarbital (#P3761, Sigma-Aldrich, USA) at 50 mg/kg body weight, prepared as a 2% solution in sterile saline. Anesthesia depth was monitored by loss of pedal withdrawal reflex. During micro-computed tomography (Micro-CT) scanning, respiration and body temperature were maintained. Immediately after imaging, while still under anesthesia, blood was collected from the retro-orbital venous plexus using non-heparinized capillary tubes. At the end of the experimental period, mice were euthanized by cervical dislocation, which was performed only by experienced researchers to ensure instantaneous death and minimize any potential pain or distress. The liver and lungs, and other tissues were collected for further analysis. All mice were housed in the SPF animal facility of the Laboratory Animal Center at Xinjiang Medical University. All animal procedures were conducted in accordance with protocols approved by the Animal Care and Use Committee of Xinjiang Medical University (Approval No. IACUC-JT-20240228-39).

### 2.11 Histopathology and immunostaining

Excised tissues were fixed, paraffin-embedded, and cut into 4-µm sections. The sections were subjected to hematoxylin and eosin (H&E) staining and immunohistochemical (IHC) staining. For IHC, tissue sections were deparaffinized, rehydrated, and underwent microwave-assisted antigen retrieval. Endogenous peroxidase activity was then blocked. The sections were incubated overnight at 4 °C with the following primary antibodies: anti-CD66b (1:100, # ab197678, Abcam, UK), anti-MPO (1:100, #66177-1-Ig, Proteintech, China), anti- CD31 (1:1000, #11265-1-AP, Proteintech, China), anti-MMP9 (1:100, #10375-2-AP, Proteintech, China), anti-VEGF (1:100, #AF5131, Affinity, China), and anti-PCNA (1:100, #AF0239, Affinity, China). This was followed by incubation with appropriate secondary antibodies at 37 °C for 30 minutes. After DAB development, counterstaining with hematoxylin, dehydration, and clearing, the sections were mounted. Stained sections were observed under a light microscope and analyzed using ImageJ software. Protein expression levels were expressed as the Average Optical Density (AOD), which was calculated as AOD = Integrated Optical Density (IOD)/Area of protein distribution.

### 2.12 Enzyme-linked immunosorbent assay

The levels of MPO-DNA, CXCL8, CXCL10 and CXCL11 in serum and cell-culture supernatants were measured using the following commercial ELISA kits: the Human MPO-DNA ELISA Kit (#ED-102148, LunChangShuo Biotech, China), Mouse MPO-DNA ELISA Kit (#ED-24993, LunChangShuo Biotech, China), Human CXCL8 ELISA Kit (#EK108, MULTI SCIENCES, China), Human CXCL10 ELISA Kit (#EK168, MULTI SCIENCES, China), and Human CXCL11 ELISA Kit (#EK1207, MULTI SCIENCES, China). The optical density (OD) was read at 450 nm using a Multiskan GO microplate reader (Thermo Fisher Scientific, USA).

### 2.13 Co-culture assay

For co-culture analysis, HCerEpiC, C-33A, SiHa, and HeLa cells (1 × 10^5^ cells/well) were seeded into the upper chamber of a 0.4 µm Transwell system, while neutrophils (5 × 10^5^ cells/well) were placed in the lower chamber of a 24-well plate and cultured for 48 hours. To inhibit CXCL8, SiHa cells were transfected with either sh-CXCL8 or sh-NC prior to co-culture under the same conditions.

### 2.14 Cell transfection

SiHa cells were transduced with lentiviral particles carrying CXCL8-specific shRNA for 24 hours and then selected with 2 μg/mL puromycin to generate stable lines. CXCL8 expression was assessed by Western blot analysis.

### 2.15 Measurement of ROS generation

Intracellular reactive oxygen species (ROS) levels were detected using the fluorescent probe DCFH-DA (#S0033, Beyotime, China). Neutrophils treated with purified CXCL8 protein (50ng/mL, # HY-P7224, MedChemExpress, USA) or Reparixin (100nmol/L, # HY-15251, MedChemExpress, USA), a CXCL8 receptor inhibitor, were divided into the following groups: None, Rosup (positive control), CXCL8 treatment, and CXCL8 + Reparixin treatment. Cells were washed with serum-free medium and resuspended in diluted DCFH-DA solution at a density of 1 × 10^6^ cells/mL. The suspension was incubated at 37 °C for 20 minutes with gentle mixing every 3~5 minutes to ensure thorough contact between the probe and cells. Subsequently, the cells were washed three times with serum-free medium to remove unincorporated probe. Fluorescence intensity was measured using a fluorescence microplate reader with an excitation wavelength of 488 nm and an emission wavelength of 525 nm.

### 2.16 Statistical analysis

Statistical analysis was performed using GraphPad Prism 10 and SPSS 27.0. Measurements were expressed as mean ± standard deviation (x̄± SD). Comparisons between two groups were made using Student’s t-test. Comparisons among three or more groups were performed using one-way ANOVA. The Kruskal-Wallis rank sum test was used when the variances were not homogeneous. Categorical data were expressed as the number of cases, and comparisons between groups were performed using the chi-square test or Fisher’s exact probability test. *P* value < 0.05 was considered statistically significant. Each mean value was derived from a minimum of three independent experiments.

## 3 Results

### 3.1 The HPV post-infection microenvironment drives neutrophil differentiation into a terminal subset with NETs-forming potential

**Figure 1:**
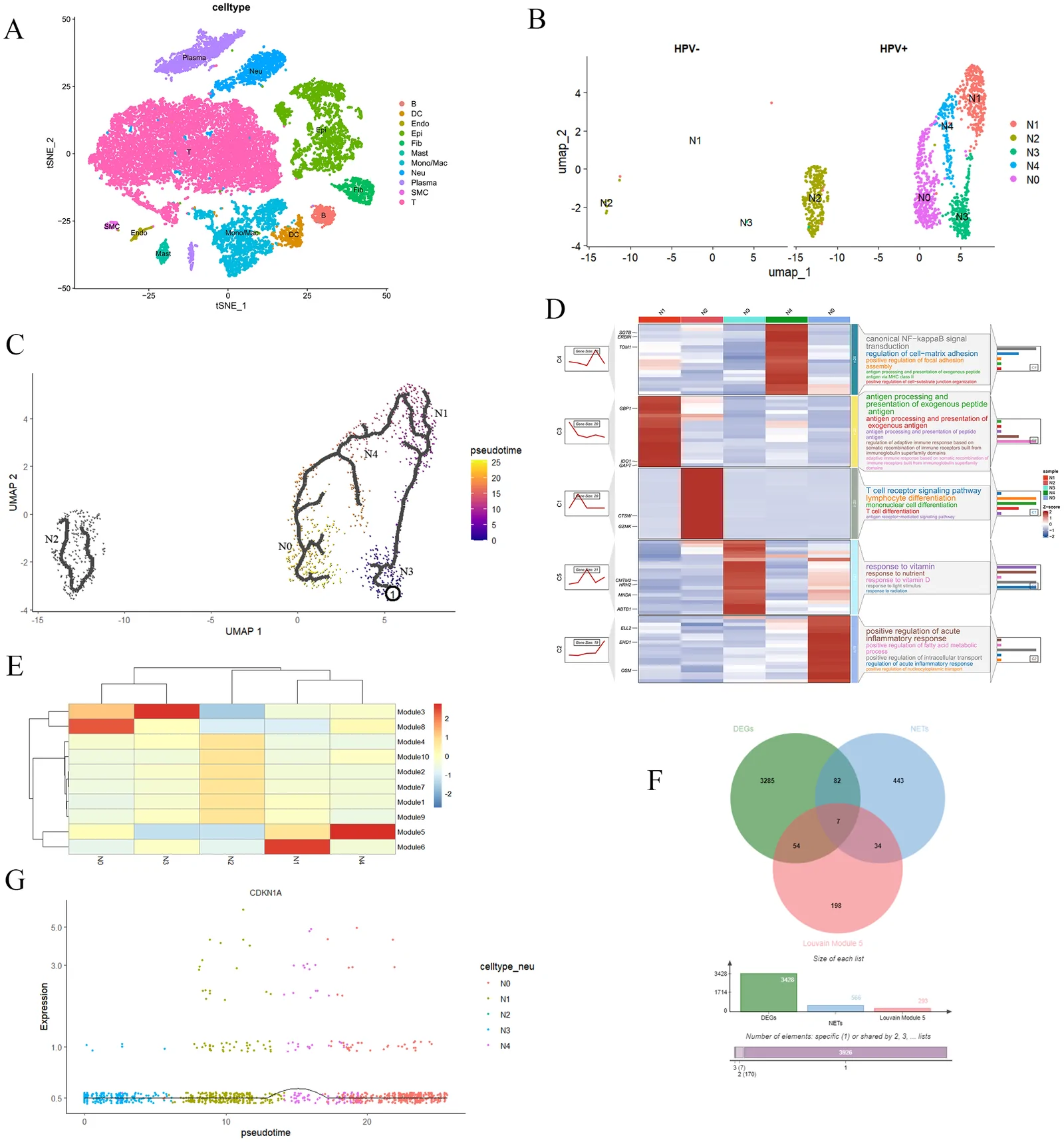
Analysis of neutrophil heterogeneity and function in the HPV-positive CC microenvironment using single-cell transcriptomics. **(A)** t-distributed stochastic neighbor embedding (t-SNE) projection of single-cell transcriptomes. **(B)** Stacked bar plot showing the relative proportions of five neutrophil functional subsets (N0-N4) in HPV-negative versus HPV-positive samples. **(C)** Single-cell developmental trajectory of neutrophils in the HPV-positive CC dataset, inferred from UMAP projection and pseudotime analysis. **(D)** Heatmap depicting functional enrichment analysis for neutrophil subsets in the HPV-positive CC cohort. **(E)** Heatmap of Louvain community detection analysis. **(F)** Venn diagram of the NETs-associated gene set, GSE151666 transcriptome data differentially expressed gene set, and neutrophil N4 subset signature gene Module 5 set (NETs: NETs-associated gene set selected from GeneCards; DEGs: GSE151666 transcriptome data differentially expressed gene set; Louvain Module 5: neutrophil N4 subset signature gene Module 5 set in HPV-positive cervical cancer). **(G)** Dynamic expression profile of the CDKN1A gene across the N0-N4 neutrophil subsets along the pseudotime trajectory.

To preliminarily explore the impact of HPV infection on neutrophil composition, we performed scRNA-seq analysis based on limited publicly available cervical cancer samples, including 2 HPV-negative cervical cancer samples from GSE197461 and 3 HPV-positive cervical cancer samples from GSE208653. Cell clusters were generated at a resolution of 0.5 and annotated into 11 major cell types-B cells, dendritic cells, endothelial cells, fibroblasts, mast cells, monocyte/macrophages, neutrophils, plasma cells, smooth muscle cells, epithelial cells, and T cells—based on canonical markers (**Figures 1A, B**). Further analysis of the neutrophil population revealed five functionally distinct subsets (N0, N1, N2, N3, N4). HPV-positive samples exhibited greater neutrophil heterogeneity and contained more unique subsets, whereas HPV-negative samples showed relatively uniform neutrophil populations with lower abundance (**Figure 1C**).

We performed trajectory inference and pseudotime analysis on neutrophils from HPV-positive samples, which indicated that neutrophil subsets differentiated starting from N3 and progressively branched into N1, N4, and N0 subsets. The N4 subset was predominantly located in the mid-to-late stage of the trajectory, coinciding with the timing of NETs formation (**Figure 1E**). Functional enrichment analysis of neutrophil subsets in HPV-positive samples revealed that the N4 subset was strongly associated with canonical NF-kappaB signal transduction, regulation of cell–matrix adhesion, and positive regulation of focal adhesion assembly (**Figure 1D**), suggesting that N4 may represent a NETs-prone neutrophil subpopulation.

Integrated Louvain-based gene module analysis identified four characteristic modules corresponding to subset identities: Module 3, 6, 8, and 5 were specifically enriched in the N3, N1, N0, and N4 subsets, respectively (**Figure 1E**). Given the critical association of the N4 subset with NETs formation, we defined Module 5-highly expressed in N4-as the core gene set representative of a transcriptional signature compatible with NET-forming potential. Taking the intersection of Module 5 genes, NETs-related genes from GeneCards, and differentially expressed genes from the bulk transcriptome dataset GSE151666 (comprising 10 HPV-negative and 58 HPV-positive CC samples), we identified seven key genes: LGALS3, ANP32B, CDKN1A, CXCR4, CD44, IL1R1, and CD68 (**Figure 1F**). Among these, the peak expression of CDKN1A was observed during the pseudotime stage occupied by the N4 subset (**Figure 1G**). CDKN1A encodes p21, a key regulator of the cell cycle that also plays important roles in immune regulation. Notably, because the scRNA-seq analysis was based on limited public samples, we consider this CDKN1A upregulation as an exploratory clue rather than a definitive mechanistic finding. We subsequently validated the role of p21 in NETosis through independent *in vitro* functional assays (shown in Section 3.7), thereby linking the transcriptomic clue to functional mechanism.

### 3.2 NETs formation is closely associated and highly expressed in HPV-positive CC

**Figure 2:**
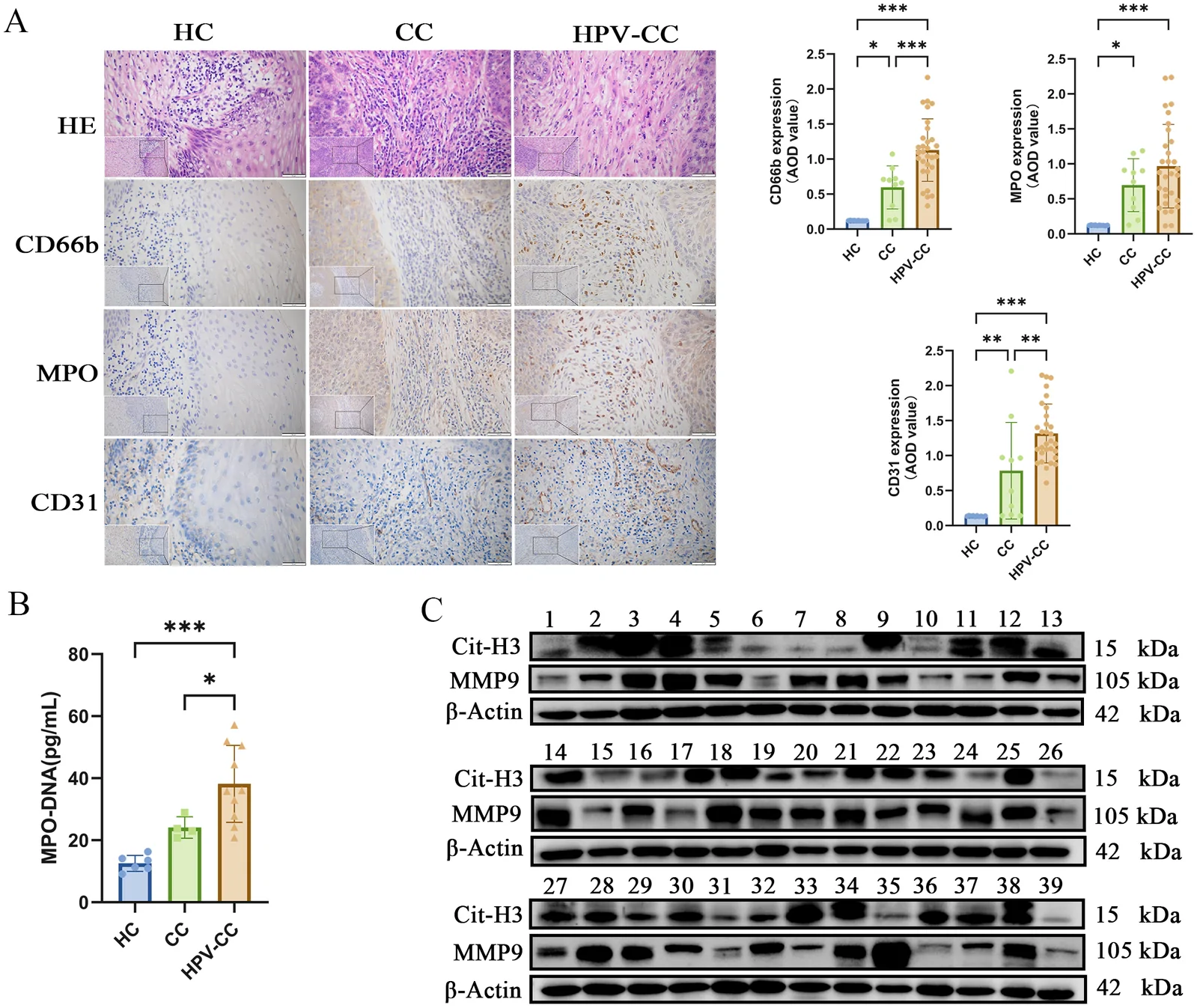
Elevated NETs levels in CC, particularly in HPV-positive CC. **(A)** Expression of CD66b, MPO, and CD31 in cervical cancer and normal cervical tissues was detected by H&E and IHC. The inset shows a representative area at 200× magnification; the main panel shows a view at 400× magnification. **(B)** Serum levels of MPO-DNA (serum marker for NETs) were measured by ELISA in CC patients and healthy controls. **(C)** Expression of Cit-H3 and MMP9 in 39 cases of HPV-positive CC tissue as detected by immunoblotting. Data were presented as mean ± SD. *p < 0.05, **P < 0.01, ***P < 0.001. HC, healthy control; CC, HPV-negative cervical cancer; HPV-CC, HPV-positive cervical cancer.

Tissues from normal subjects (n=10), HPV-negative CC patients (n=10), and HPV-positive CC patients (n=30) were analyzed by IHC for expression of the tumor-associated neutrophil marker CD66b, the NETs marker MPO, and the vascular marker CD31. CD66b and MPO were localized in the interstitial cytoplasm and exhibited spatial co-localization (**Figure 2A**) (**Supplementary Figure 1**). CD31 was localized in the cytoplasm of endothelial cells within interstitial microvessels. The number of CD66b-positive neutrophils was higher in both HPV-negative and HPV-positive CC tissues than in normal cervical tissues and was significantly greater in HPV-positive cancers than in HPV-negative cancers. NETs abundance and blood vessel density were both elevated in CC tissues, with significantly higher levels in HPV-positive patients than in HPV-negative patients (**Figure 2A**). Additionally, serum levels of MPO-DNA, a circulating NETs marker, were elevated in CC patients, particularly in those with HPV-positive disease (**Figure 2B**).

Furthermore, Western blot analysis of 39 HPV-positive CC tissues revealed expression of Cit-H3 and MMP9 (**Figure 2C**). Based on the mean expression level, cases were categorized into high- and low-expression groups for correlation with clinicopathological parameters. High Cit-H3 expression was associated with larger tumor diameter, deep stromal invasion, and poor differentiation. High MMP9 expression was correlated with deep stromal invasion, suggesting an association with poor prognosis (**Table 1**). To identify potential prognostic factors, we performed univariate analysis of 5-year survival rates in 39 cervical cancer patients (**Table 2**). Among the clinicopathological parameters, age ≤45 years was associated with a significantly lower 5-year survival rate compared with age >45 years. Lymph node metastasis was also significantly associated with worse survival. In addition, high Cit-H3 expression was significantly correlated with a reduced 5-year survival rate. No significant associations were observed for FIGO stage, tumor size, depth of invasion, vascular invasion, pathological grading, pathological type, or MMP9 expression. To determine whether the above significant factors were independent predictors of prognosis, we performed multivariate Cox regression analysis incorporating age, lymph node metastasis, and Cit-H3 expression (**Table 3**). The results showed that Cit-H3 expression remained an independent prognostic factor for 5-year survival. However, age and lymph node metastasis did not retain statistical significance in the multivariate model.

**Table 1:** Relationship between Cit-H3 and MMP9 expression and clinicopathologic features in 39 HPV-positive cervical cancer patients.

Features	n	Cit-H3 expression				MMP9 expression			
		High	Low	χ²	*P* value	High	Low	χ²	*P* value
Age (years)									
≤45	18	10	8	0.039	0.843	9	9	0.022	0.882
>45	21	11	10			10	11		
FIGO stage									
I	20	9	11	1.293	0.256	10	10	0.027	0.869
II-III	19	12	7			9	10		
Tumor size									
≤4cm	17	6	11	4.174	0.041	6	11	2.174	0.14
>4cm	22	15	7			13	9		
Lymph node metastasis									
No	29	14	15	–	0.235	13	16	–	0.408
Yes	10	7	3			6	4		
Depth of invasion									
≤1/2	16	4	12	9.084	0.003	4	12	6.109	0.013
>1/2	23	17	6			15	8		
Vascular invasion									
No	20	8	12	3.167	0.075	8	12	1.249	0.264
Yes	19	13	6			11	8		
Pathological grading									
Middle differentiation	23	9	14	4.885	0.027	10	13	0.616	0.433
Low differentiation	16	12	4			9	7		
Pathological type									
Squamous carcinoma	35	20	15	–	0.079	19	16	–	0.059
Adenocarcinoma	3	0	3			0	3		
Adenosine squamous carcinoma	1	1	0			0	1		

**Table 2:** Univariate analysis of 5-year survival rate in cervical cancer patients cancer.

Features	n	Death or recurrence within 5 years	Overall survival rate (%)	*χ²*	P value
Age (years)					
≤45	18	10	44.44	5.614	0.018
>45	21	4	80.95		
FIGO stage					
I	20	8	60.00	0.3	0.584
II-III	19	6	68.42		
Tumor size					
≤4cm	17	4	76.47	2.003	0.157
>4cm	22	10	54.55		
Lymph node metastasis					
No	29	7	75.86	–	0.019
Yes	10	7	30.00		
Depth of invasion					
≤1/2	16	4	75.00	1.4	0.237
>1/2	23	10	56.52		
Vascular invasion					
No	20	10	50.00	3.548	0.06
Yes	19	4	78.95		
Pathological grading					
Middle differentiation	23	6	73.91	2.345	0.126
Low differentiation	16	8	50.00		
Pathological type					
Squamous carcinoma	35	12	65.71	–	1
Adenocarcinoma	3	0	100.00		
Adenosine squamous carcinoma	1	1	0.00		
Cit-H3 expression					
High	21	11	47.62	5.373	0.02
Low	18	3	83.33		
MMP9 expression					
High	19	6	68.42	0.30	0.58
Low	20	8	60		

**Table 3:** Multivariate Cox regression analysis of prognosis in cervical cancer patients.

Variables	β	SE	HR (95% CI)	*P* value
Age	0.847	0.646	2.332 (0.657, 8.274)	0.19
Lymph node metastasis	0.625	0.601	1.869 (0.576, 6.065)	0.298
Cit-H3 expression	1.356	0.671	3.881 (1.042, 14.458)	0.043

### 3.3 NETs promote malignant growth and tumor angiogenesis in CC

**Figure 3:**
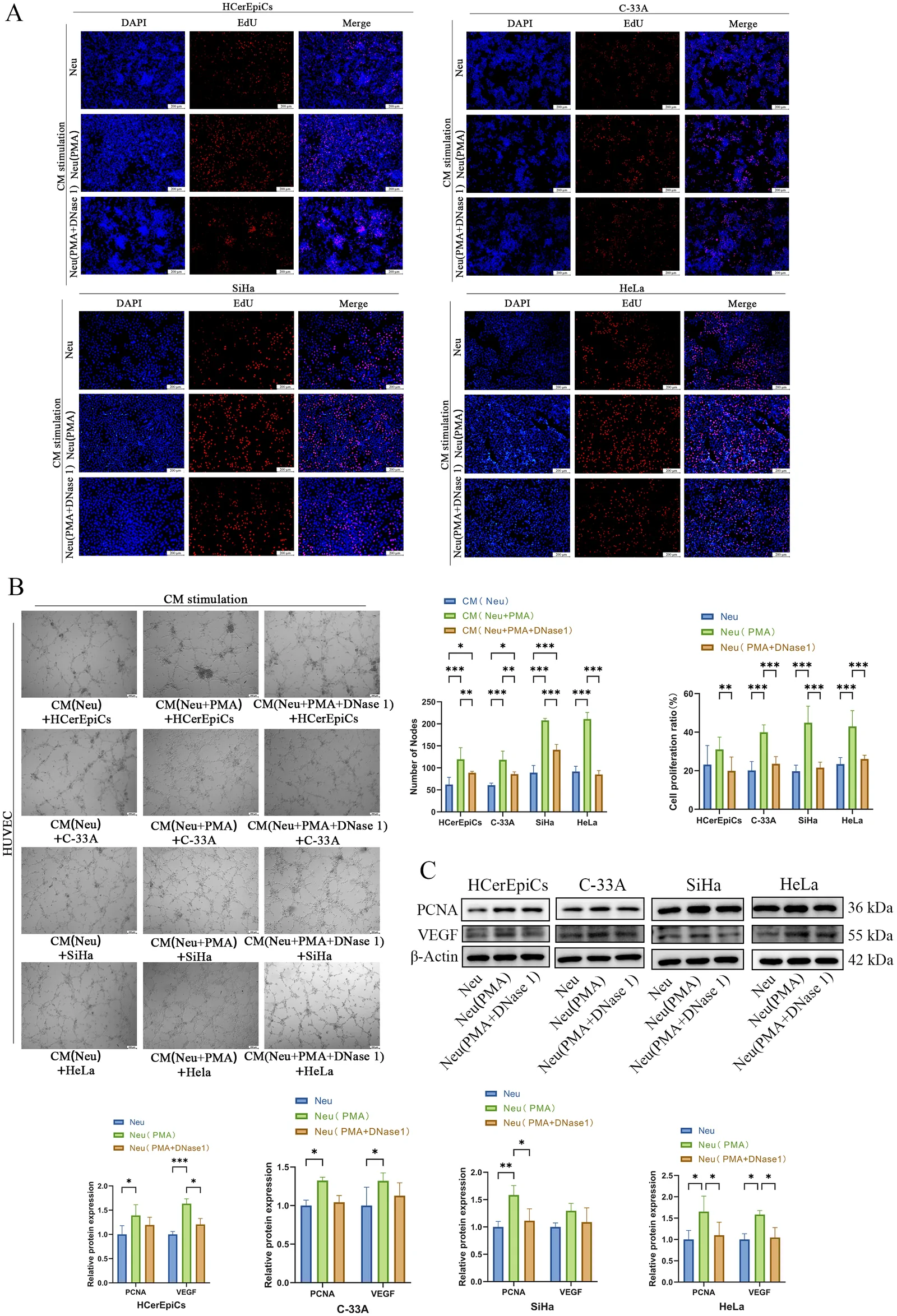
NETs promote malignant growth and tumor angiogenesis in CC. **(A)** EdU assays were performed after HCerEpiC, C-33A, SiHa, and HeLa cells had been exposed for 24 hours to CM(Neu) derived from untreated neutrophils, to CM from PMA-stimulated neutrophils (Neu+PMA), or to CM from PMA-stimulated neutrophils and treated with DNase 1 (Neu+PMA+DNase 1). **(B)** Tube-formation assays were carried out on Matrigel for 6 hours using HUVECs incubated with CM collected from HCerEpiC, C-33A, SiHa, and HeLa cells that had been pre-treated for 48 hours with CM(Neu), CM (Neu + PMA), or CM (Neu + PMA + DNase 1). **(C)** Western blot analysis of PCNA and VEGF expression in HCerEpiC, C-33A, SiHa, and HeLa cells after 48 hours of exposure to CM(Neu), CM (Neu + PMA), or CM (Neu + PMA + DNase 1). Densitometric values were normalized to β-actin and expressed relative to the control group. Data are presented as mean ± SD. *p < 0.05, **P < 0.01, ***P < 0.001.

Due to the limited yield and short ex vivo lifespan of neutrophils isolated from human peripheral blood, which constrain functional studies, we differentiated HL-60 cells into neutrophil-like cells using 1 μmol/L ATRA for subsequent experiments (**Supplementary Figure 2A**). PMA is a classical inducer of NETs formation, and DNase 1 is a NETs inhibitor (**Supplementary Figure 2B**). To assess the impact of NETs on CC progression, normal cervical epithelial cells (HCerEpiC), HPV-negative CC cells (C-33A), and HPV-positive CC cells (SiHa and HeLa) were exposed to CM derived from neutrophils treated with PMA in the presence or absence of DNase 1. CM (Neu + PMA) significantly promoted proliferation in all four cell lines compared with CM (Neu) or CM (Neu + PMA + DNase 1), with the most pronounced enhancement in SiHa and HeLa cells (**Figure 3A**). Consistently, Western blot analysis revealed elevated expression of the proliferation marker PCNA in these cells. To investigate whether NETs could regulate tumor angiogenesis, we collected CM from the four cell lines treated as described above and used it to stimulate HUVECs, and subsequent tube formation was assessed. CM from cells treated with CM (Neu + PMA) resulted in enhanced tube formation by HUVECs, an effect that was partially abolished when NETs were degraded by CM (Neu + PMA + DNase 1) (**Figure 3B**). Furthermore, increased expression of the pro-angiogenic factor VEGF was detected in cells treated with CM (Neu + PMA) (**Figure 3C**), suggesting that NETs may promote angiogenesis by upregulating VEGF expression in CC cells.

### 3.4 NETs promote CC growth *in vivo*

**Figure 4:**
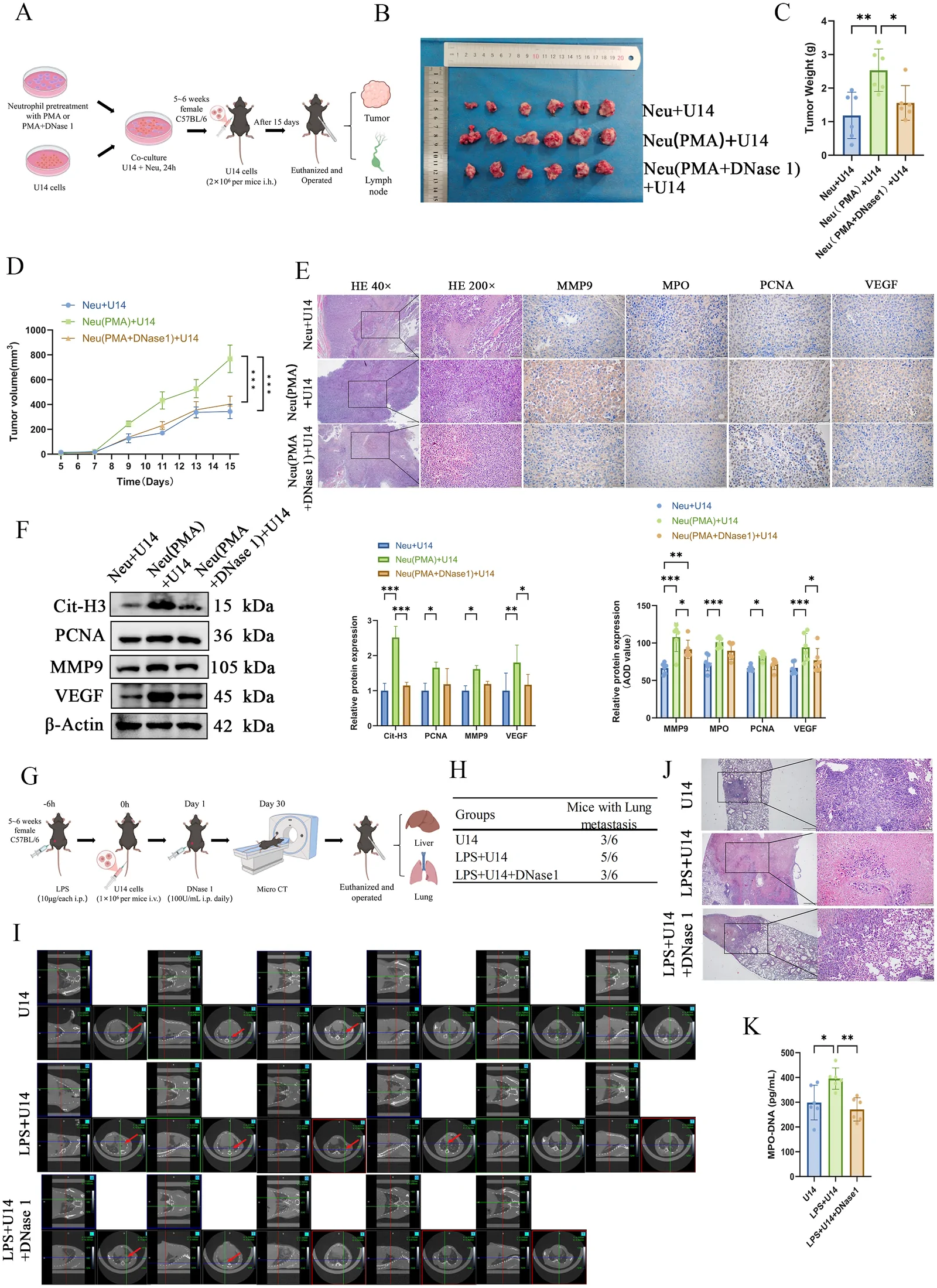
NETs promote the growth and metastasis of CC *in vivo*. **(A)** Schematic diagram of the subcutaneous xenograft model of CC in mice. **(B)** Representative images of tumor-bearing mice from the Neu+U14, Neu (PMA)+U14, and Neu (PMA+DNase 1) +U14 co-injection groups (n=6 per group). **(C)** Tumor weight (g) for each group. **(D)** Tumor growth curves for each group. **(E)** H&E staining and IHC staining for MMP9, MPO, PCNA, and VEGF in tumor tissues (400×). **(F)** Western blot analysis of MMP9, Cit-H3, PCNA, and VEGF expression. **(G)** Schematic diagram of the mouse CC tail vein metastasis model. **(H)** Number of mice with lung metastases after 30 days. **(I)** Representative micro-CT images of lungs from mice in the U14, LPS+U14, and LPS+U14+DNase 1 groups (red arrows indicate metastatic lesions). **(J)** Lung metastases detected by H&E staining in each group (left panel: 40× magnification; right panel: 200× magnification). **(K)** Serum levels of MPO-DNA complexes in mice from each group were measured by ELISA. Data are presented as mean ± SD. *p < 0.05, **P < 0.01, ***P < 0.001. i.h, hypodermic injection; i.p, intraperitoneal injection; i.v, intravenous injection; LPS, lipopolysaccharide.

To determine the impact of NETs on cervical cancer growth *in vivo*, C57BL/6 female mice were subcutaneously injected with U14 cells that had been co-cultured with neutrophils for 24 hours. At the time of injection, neutrophils had already undergone apoptosis and released NETs, thus no human-derived neutrophils will appear in immunocompetent C57BL/6 female mice. The PMA group markedly accelerated tumor outgrowth, whereas DNase 1-mediated degradation of NETs largely reversed this effect (**Figures 4A–D**), body weight changes in the respective groups followed a similar trend (**Supplementary Figure 2C**). Immunostaining revealed strong up-regulation of the metastasis marker MMP9, the NETs marker MPO, the proliferation index proliferating cell nuclear antigen (PCNA), and the pro-angiogenic factor vascular endothelial growth factor (VEGF) in tumors from the Neu (PMA) + U14 group; each of these signals was appreciably reduced when NETs were disrupted by DNase 1 (**Figure 4E**). Western blot analysis corroborated these findings at the protein level (**Figure 4F**). Additionally, lymph node metastasis was observed in all experimental groups, with varying degrees of severity (**Supplementary Figure 2D**). Collectively, the data indicate that NETs foster an intratumoral milieu that enhances proliferation, invasion, metastasis, and neovascularization of U14 CC cells *in vivo*, and that enzymatic dissolution of NETs effectively attenuates this tumor-promoting program.

### 3.5 NETs promote invasion and migration of CC cells

**Figure 5:**
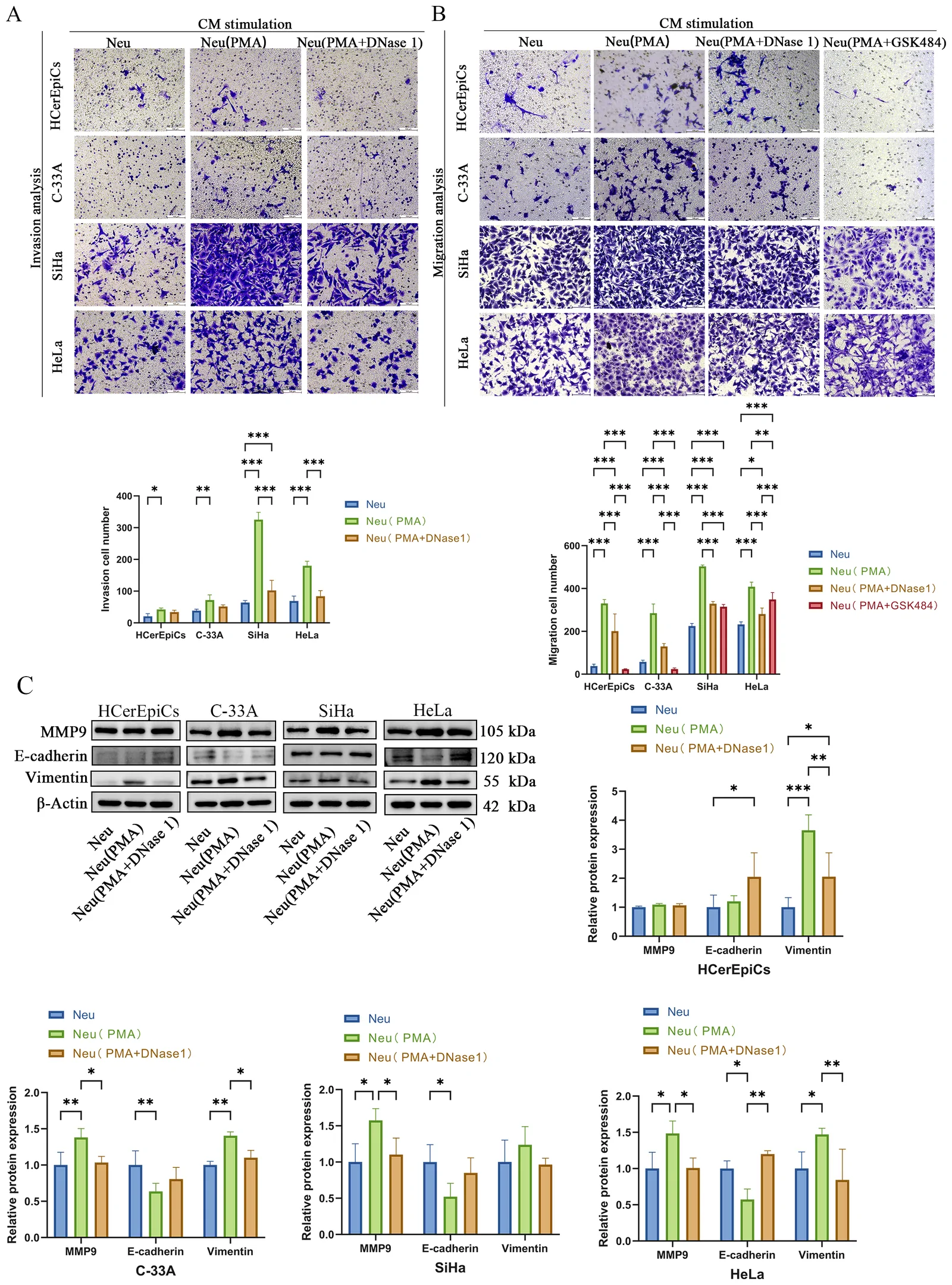
NETs promote invasion and migration of CC cells. **(A)** Transwell invasion assay showing cell invasion in HCerEpiC, C-33A, SiHa, and HeLa cells after 48 hours treatment with CM from (Neu), (Neu + PMA), or (Neu + PMA + DNase 1). **(B)** Cell migration of HCerEpiC, C-33A, SiHa, and HeLa cells after 48 hours treatment with CM(Neu), CM(Neu + PMA), or CM(Neu + PMA + DNase 1), and CM(Neu+PMA+GSK484). **(C)** Western blot analysis of MMP9, E-cadherin, and vimentin expression in HCerEpiC, C-33A, SiHa, and HeLa cells treated with different CM for 48 hours. Density ratios were normalized to β-actin and compared to the control group. Data are presented as mean ± SD. *p < 0.05, **P < 0.01, ***P < 0.001.

Cell-invasion and migration assays were performed after exposing each cervical-cancer line to the indicated CM. CM from PMA-stimulated neutrophils CM(Neu+PMA) markedly increased the number of invading cells; the effect was most pronounced in SiHa and HeLa cells, whereas CM prepared in the presence of DNase 1 CM(Neu+PMA+DNase 1), which disrupts NETs, attenuated invasion (**Figure 5A**). Transwell migration and invasion assays consistently showed that CM from PMA-stimulated neutrophils significantly promoted migration and invasion of cervical cancer cells, particularly in HPV-positive lines, whereas this effect was abrogated by either DNase 1-mediated NET degradation or PAD4 inhibition with GSK484 (**Figure 5B**). Western blot analysis revealed that CM(Neu+PMA) up-regulated the invasion-related metalloproteinase MMP9 in all tested cancer cells. To evaluate epithelial–mesenchymal transition (EMT), we probed for E-cadherin and vimentin, E-cadherin reduced while vimentin increased in CM(Neu+PMA) (**Figure 5C**).

### 3.6 NETs promote experimental lung colonization of CC cells *in vivo*

To further define the impact of NETs on the metastatic behavior of CC cells *in vivo*, C57BL/6 female mice were primed with LPS to elicit NETs formation, followed by tail-vein inoculation of U14 cells to establish a hematogenous metastasis model (**Figure 4G**). Thirty days later, Micro-CT was performed before necropsy and histopathological examination. We monitored mouse body weight to assess overall health status (**Supplementary Figure 2E**). Compared with the control group, combined LPS and U14 treatment significantly suppressed body weight gain. However, disruption of NETs by DNase 1 largely reversed this suppressive effect, restoring body weight growth to a level comparable to that of the control group. Micro-CT images and H&E staining revealed pulmonary metastatic foci. Integrating both modalities, we observed that LPS-induced NETs increased the incidence of lung metastasis, whereas DNase 1-mediated degradation of NETs mitigated this effect (**Figures 4H–J**). Serum NET levels, quantified by ELISA, were higher in the LPS+U14 group than in the U14 group, and this elevation was abolished by DNase 1 treatment (**Figure 4K**). No evident hepatic metastases were detected in any group (**Supplementary Figure 2F**).

### 3.7 HPV promotes NETs formation via the CXCL8-CXCR1/2 -ROS axis

**Figure 6:**
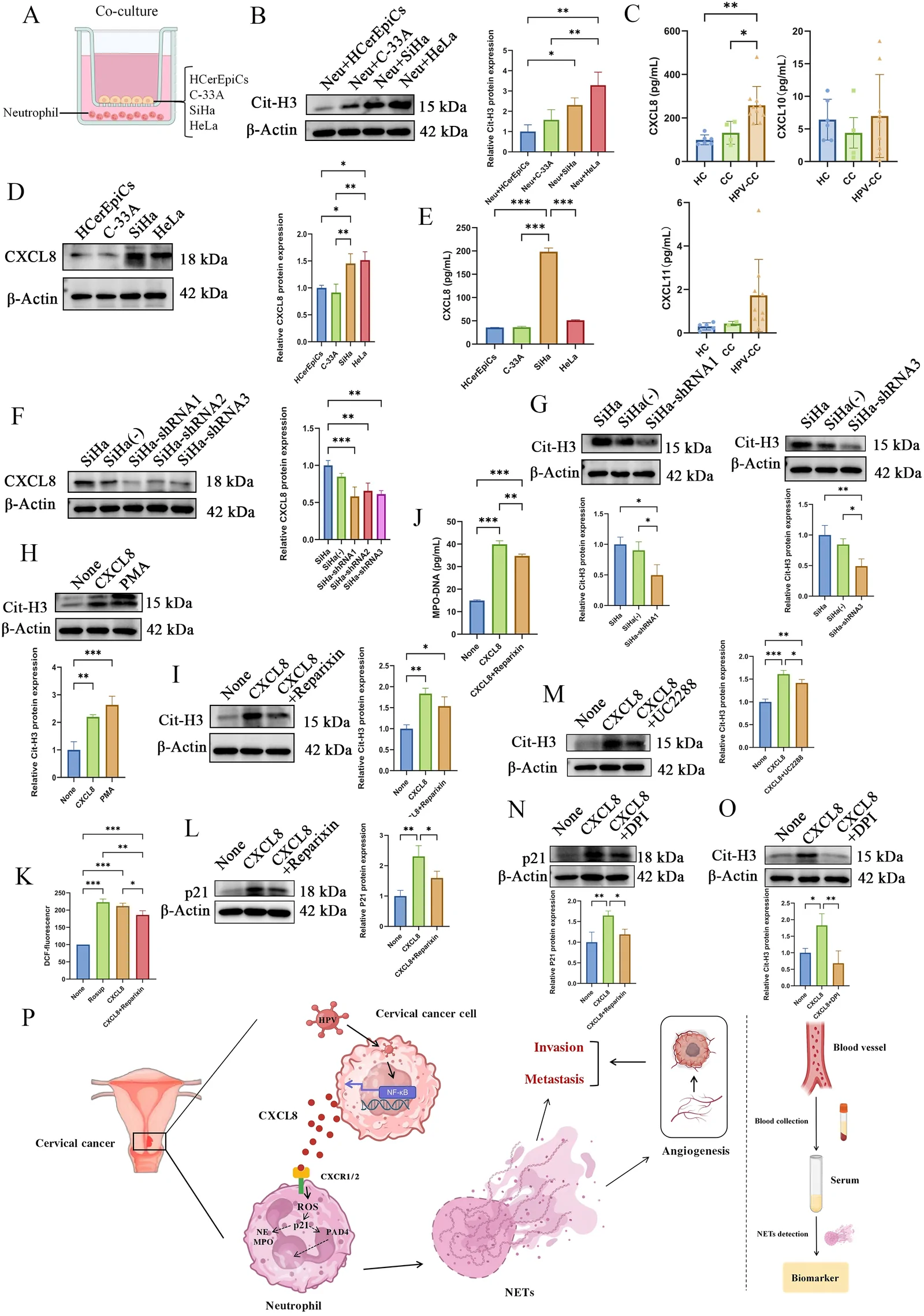
HPV promotes NETs formation via the CXCL8-CXCR1/2-ROS axis. **(A)** Schematic diagram of the co-culture system for analyzing NETs formation after 48-hour co-culture of neutrophils with normal cervical epithelial cells (HCerEpiC), HPV-negative cervical cancer cells (C-33A), or HPV-positive cervical cancer cells (SiHa and HeLa). **(B)** Western blot analysis of Cit-H3 expression in neutrophils after 48-hour co-culture with HCerEpiC, C-33A, SiHa, or HeLa cells. **(C)** Serum levels of CXCL8, CXCL10, and CXCL11 in healthy individuals, HPV-negative, and HPV-positive CC patients were measured by ELISA. **(D)** Western blot analysis of baseline CXCL8 expression in HCerEpiC, C-33A, SiHa, and HeLa cells. **(E)** Secreted CXCL8 levels in the supernatant of HCerEpiC, C-33A, SiHa, and HeLa cells were measured by ELISA. **(F)** Western blot analysis of CXCL8 protein expression in SiHa cells transduced with lentivirus carrying sh-NC or sh-CXCL8. **(G)** Western blot analysis of Cit-H3 expression in neutrophils after co-culture with SiHa cells transduced with lentivirus carrying sh-RNA1 or sh-RNA3. **(H)** Western blot analysis of Cit-H3 in neutrophils treated for 12 hours with or without purified CXCL8 protein (50 ng/mL) or PMA (25 nmol/L). **(I)** Western blot analysis of Cit-H3 in neutrophils treated for 12 hours with or without purified CXCL8 protein or Reparixin (100 nmol/L). **(J)** ELISA of MPO-DNA complexes in the supernatant of neutrophils treated for 12 hours with or without purified CXCL8 protein or Reparixin. **(K)** ROS analysis using DCFH-DA staining in neutrophils treated for 12 hours with Rosup (a ROS-positive control reagent), CXCL8, or Reparixin. **(L)** Western blot analysis of p21 in neutrophils treated for 12 hours with or without purified CXCL8 protein or Reparixin. **(M)** Western blot analysis of Cit-H3 in neutrophils treated for 12 hours with or without purified CXCL8 proteinor UC2288 (2.5 μmol/L). **(N, O)** Western blot analysis of p21 and Cit-H3 in neutrophils treated for 12 hours with or without purified CXCL8 protein or DPI (10 μmol/L). β-Actin was used as a loading control. **(P)** Schematic mechanism of this study: HPV-induced CC cells secrete abundant CXCL8 to recruit neutrophils; CXCL8 then activates the CXCR1/2-ROS-p21 signaling axis in these neutrophils, leading to NETs formation that drives angiogenesis and malignant progression of CC. In addition, monitoring circulating NETs may have the potential to serve as a biomarker for predicting the prognosis of HPV-associated CC. HPV, human papillomavirus; NE, neutrophil elastin; MPO, myeloperoxidase; PAD4, peptide arginine deaminase 4; CXCR, CXC-chemokine receptor. Data are presented as mean ± SD. *p < 0.05, **P < 0.01, ***P < 0.001.

Neutrophils were co-cultured for 48 h with HCerEpiC, C-33A, SiHa and HeLa, and NETs formation was subsequently quantified (**Figure 6A**). Compared with HCerEpiC, all CC cell lines elicited a marked increase in NETs release; this effect was most pronounced when neutrophils were exposed to HPV-positive SiHa and HeLa cells (**Figure 6B**), suggesting that CC cells particularly following HPV infection, secrete soluble factors that drive NETosis, the process of NETs formation.

To identify candidate mediators, we performed transcriptome sequencing on fresh tumor tissues from three HPV-positive and three HPV-negative cervical cancers. Among the differentially expressed genes, the alarmin chemokines CXCL8, CXCL10, and CXCL11 were identified as candidates for further investigation. After expanding the cohort, serum ELISA revealed that CXCL8 was significantly elevated in HPV-positive patients (**Figure 6C**). Baseline CXCL8 expression was next examined in the four cell lines. Western blot revealed high intracellular CXCL8 in HPV-positive cells, and ELISA of culture supernatants showed the highest CXCL8 levels in SiHa cells (**Figures 6D, E**). To determine whether CXCL8 is functionally required for NETs induction, SiHa cells were transduced with lentiviral shRNA targeting CXCL8 (**Figure 6F**). Co-culture of CXCL8-depleted SiHa with neutrophils resulted in a significant reduction in NETs formation (**Figure 6G**).

We then asked whether CXCL8 acts directly on neutrophils. Purified CXCL8 triggered NETs release to an extent comparable with the classic inducer PMA (**Figure 6H**). This effect was abolished when neutrophils were pre-treated with the selective CXCR1/2 antagonist Reparixin (**Figures 6I, J**), implicating CXCR1/2 as the requisite receptors. Since ROS are canonical inducers of NETosis, we measured intracellular ROS after CXCL8 or Reparixin exposure. CXCL8 increased ROS generation to levels similar to the positive control Rosup, whereas Reparixin markedly attenuated both ROS accumulation and NETs formation (**Figure 6K**), confirming that ROS operate downstream of CXCL8–CXCR1/2 axis during HPV-driven NETosis. Finally, prompted by single-cell transcriptomic analysis suggesting a key role for p21 in NETs formation, we validated its protein-level expression by Western Blot. The results demonstrated that CXCL8 significantly promoted p21 protein expression, an effect inhibited by the CXCR1/2 antagonist Reparixin (**Figure 6L**). Furthermore, the p21 inhibitor UC2288 attenuated CXCL8-induced Cit-H3 expression in neutrophils (**Figure 6M**). Collectively, these functional assays provide evidence that p21 may act as a downstream candidate molecule. To determine whether ROS acts upstream of p21, we pre-treated neutrophils with the NADPH oxidase inhibitor DPI prior to CXCL8 stimulation. DPI pretreatment significantly decreased p21 expression and Cit-H3 levels (**Figures 6N,O**). These findings indicate that ROS functions upstream of p21 in the CXCL8-induced NETosis pathway. To investigate the biological function of CXCL8 in cervical cancer, we analyzed the TCGA-CESC dataset. Survival analysis revealed that high CXCL8 expression was significantly associated with poor prognosis (**Supplementary Figure 3A**). Immune infiltration analysis showed that CXCL8 expression correlated with the infiltration levels of neutrophils, mast cells, and dendritic cells in the cervical cancer microenvironment. Notably, CXCL8 exhibited a strong positive correlation with neutrophil infiltration (*P* < 0.01) (**Supplementary Figures 3B, C**). Furthermore, we compared the differences in chemotherapy and drug sensitivity between CXCL8 high- and low-expression groups. The results showed that patients with low CXCL8 expression were more sensitive to multiple chemotherapeutic agents and immunosuppressants, suggesting that CXCL8 may recruit neutrophils to establish an immunosuppressive microenvironment, thereby enhancing drug resistance in cervical cancer patients (**Supplementary Figure 3D**).

## 4 Discussion

CC remains one of the most common malignancies in women worldwide, with persistent high-risk HPV infection being its primary cause. Although the oncogenic mechanisms of HPV have been extensively studied, the tumor immune microenvironment shaped by HPV infection, particularly the role of neutrophils, remains incompletely understood. This study focused on NETs, a novel immune effector mechanism, to systematically elucidate their function in CC progression and their potential association with HPV infection. To investigate the impact of HPV infection on neutrophil in the CC microenvironment, we performed an integrated analysis of single-cell RNA sequencing data from the GEO database. Cell clustering analysis identified 11 major cell populations, including a highly heterogeneous neutrophil population. Further subclustering of neutrophils revealed five functionally distinct subsets (N0-N4). HPV-positive samples exhibited significantly heightened neutrophil diversity and abundance, suggesting that HPV infection may drive functional reprogramming of neutrophils in the tumor microenvironment. Pseudotime trajectory analysis revealed a developmental path initiating from the N3 subset and branching toward N1, N4 and N0 subsets. Notably, the N4 subset emerged predominantly at intermediate-to-late pseudotime stages, representing mid-to-late neutrophil differentiation, temporally aligned with NETs formation. Functional enrichment analysis further supported this subset as the key population with neutrophil extracellular trap-forming potential. Based on these findings, we hypothesize that the HPV-associated microenvironment induces neutrophil differentiation into a neutrophil extracellular trap-prone N4 subset, thereby promoting neutrophil extracellular trap formation. However, this study only collected single-cell sequencing data from 2 HPV-negative samples in GSE197461 and 3 HPV-positive samples in GSE208653, which represented all samples meeting the inclusion criteria for single-cell sequencing in HPV-positive and HPV-negative CC currently available in public databases, and the sample size is relatively limited. Nevertheless, these results still suggest a potential association between NETs and HPV-positive CC, providing direction for subsequent research. To address this limitation, we performed validation studies using clinical samples.

To this end, we examined NET-related markers in clinical tissues and serum. Our data demonstrate significant enrichment of NETs in CC tissues. IHC analysis revealed upregulation of the neutrophil marker CD66b, MPO and the tumor vascular marker CD31 in CC tissues, with higher expression levels observed in HPV-positive cases. These morphological findings suggest that activated neutrophils may undergo NETosis within the tumor microenvironment. This histological evidence is further supported by serological data: the serum levels of MPO-DNA complexes, a specific circulating marker of NETs, were significantly elevated in CC patients, particularly in HPV-positive individuals. While confirming the presence of NETs is an essential first step, elucidating their clinical significance is of greater importance. Through immunoblotting analysis of 39 cases of HPV-positive CC tissues, we quantified the specific NETs marker Cit-H3 and MMP9, also metastasis-associated protein. The results showed that high Cit-H3 expression was significantly correlated with larger tumor diameter, deep stromal invasion, and poorer differentiation, while high MMP9 expression was relevant to deep stromal invasion. Our clinical data further support the prognostic relevance of NETs in cervical cancer. Univariate analysis revealed that high Cit-H3 expression, was significantly associated with poorer 5-year survival, and this association remained independent in multivariate Cox regression analysis. These findings directly link NETs to aggressive clinicopathological features in HPV-positive CC, suggesting that NETs are not merely an epiphenomenon but may actively drive tumor proliferation, local invasion and dedifferentiation. Our findings are consistent with the reports in other cancers, hinting that the pro-tumorigenic role of NETs is not unique to CC. Zhang et al. demonstrated that dense NETs infiltration was associated with shorter overall and disease-free survival in patients with esophageal carcinoma (20). Similarly, Okamoto et al. observed that high NETs expression predicted reduced relapse-free survival in colorectal cancer and that pharmacologic blockade of NETs formation effectively attenuated cancer cell migration (21). This collective evidence supports the view that NETs are key promoters of tumor progression rather than mere biomarkers.

NETs were initially identified as a host defense mechanism capable of trapping and eliminating pathogens and were later implicated in inflammatory diseases (22, 23). Accumulating evidence indicates that various cancer cells can induce NETs formation, thereby promoting cancer progression, a phenomenon observed in multiple solid tumors including gastric cancer, colorectal cancer, breast cancer and lung cancer (24–27). Consistent with these findings, our study confirms that CC cells similarly stimulate NETs generation, which in turn promotes malignant phenotypes through multiple mechanisms. Integrated *in vitro* and *in vivo* analysis reveal that NETs propel CC progression through a dual, synergistic mechanism. EdU assays showed that NETs markedly increased cell proliferation, which was amplified in tumor-bearing mice, where NETs treatment steepened subcutaneous tumor growth curves and elevated PCNA. DNase 1 consistently reversed the proliferative advantage, demonstrating that intact NET structures are essential for its bioactivity. Concurrently, NETs up-regulated tumor-cell VEGF expression and intensified endothelial tube formation, thereby establishing a pro-angiogenic milieu. In murine xenografts, NETs raised intratumoral VEGF levels, whereas DNase 1 abrogated these changes. These observations mirror NETs-driven angiogenesis reported in gastric cancer (28) and collectively establish that NETs coordinate proliferative and angiogenic programs to deliver a convergent growth stimulus. Collectively, these data indicate that NETs provide a synergistic growth advantage by simultaneously activating proliferative and angiogenic programs.

Effective dissemination and distant seeding represent another critical facet of malignant progression. We found that NETs robustly enhanced the invasive and migratory capacity of CC cells by inducing EMT and markedly increasing MMP9 expression, a pattern that aligns with NET-driven EMT reported in other tumor types (25, 29, 30). To further validate the specificity of NET-mediated effects on tumor invasion, we employed GSK484, a selective PAD4 inhibitor, to block NETosis. Conditioned medium from PMA-stimulated neutrophils pre-treated with GSK484 failed to promote cervical cancer cell invasion, confirming that the pro-invasive activity is specifically dependent on PAD4-mediated NET formation rather than other neutrophil-derived soluble factors. In our tail-vein lung colonization model, LPS-triggered NET formation similarly led to a significant rise in pulmonary metastatic foci. MMP9 serves as not only as an intrinsic component of NETs but also as a molecule up-regulated in tumor cells by NETs (31), this dual source provides a compelling explanation for the observed clinical association between high NETs levels and deep myometrial invasion. These findings collectively establish NETs as a core orchestrator of CC progression through their multifaceted effects on the proliferation, angiogenesis, and invasion. NETs enhance lung colonization of circulating tumor cells under tail-vein-injection conditions, while its role in spontaneous metastasis still needs further verification using orthotopic-tumor-bearing models.

In chronic inflammatory microenvironment contexts, research focused on NETs formation mechanisms has expanded from exogenous pathogen triggers to the intricate regulation by endogenous signals within tumor microenvironment (22, 32). In persistently infected microenvironments, damage-associated molecular patterns (DAMPs) secreted by various cells recruit neutrophils and bind to pattern recognition receptors on their surface (33), igniting the downstream cascades that culminate in ROS burst and NETs deployment. Studies in ovarian carcinoma, glioma and other solid tumors have shown that neoplastic cells themselves secrete factors such as G-CSF and HMGB1 that actively attract and prime neutrophils, thereby inducing NETs formation to propel tumor progression (34, 35). Based on the above, we hypothesized that a similar tumor cell-dominated endogenous NETs induction mechanism might exist in HPV-associated CC. Our results confirmed that HPV-positive CC cells indeed exhibited a significantly enhanced ability to induce NETs, correlated with increased expression and secretion of the key chemokine CXCL8. ELISA results showed that SiHa cells secreted higher levels of CXCL8, which may suggest a potential association between the alarmin CXCL8 and specific HPV types, although further experiments using a broader panel of HPV-positive cell lines are needed to address this question. To verify the necessity of CXCL8, we employed shRNA knockdown technology and demonstrated that reducing CXCL8 expression significantly attenuated the ability of HPV-positive CC cells to induce NETs formation. Further mechanistic studies revealed that CXCL8 activates the downstream signaling by binding to its specific receptors CXCR1/2—the CXCR1/2-specific inhibitor Reparixin completely blocked CXCL8-induced NETs formation. DCFH-DA assays confirmed that CXCL8 stimulation significantly increased intracellular ROS levels in neutrophils, an effect also inhibited by Reparixin, an inhibitor of CXCR1/2. Based on Louvain gene module analysis, we identified four characteristic gene modules corresponding to neutrophil subpopulation states. Among these, Module 5 was specifically enriched in the N4 subpopulation characterized by neutrophil extracellular trap formation. By intersecting the Module 5 gene set with known neutrophil extracellular trap-associated gene sets and differentially expressed genes, we identified seven key candidate genes. Notably, the expression peak of CDKN1A coincided precisely with the pseudotime differentiation stage where the N4 subpopulation resides, suggesting its encoded protein p21 may play a critical role in enabling neutrophils to acquire neutrophil extracellular trap-forming capacity. This finding prompted further investigation into the potential role of p21 in neutrophil functional regulation. As a cyclin-dependent kinase inhibitor, p21 plays central regulatory roles in cell cycle arrest, DNA damage repair, and cellular differentiation (36). Recent studies have revealed that p21 can recruit macrophages through generation of the p21-activated secretory phenotype, and when persistently expressed, drives macrophage polarization toward the M1 phenotype (37). Additionally, research has demonstrated that reactive oxygen species, as signaling molecules, can increase p21 expression through checkpoint kinase 2, thereby inducing cell cycle arrest and subsequent M1 macrophage polarization (38). These findings indicate that p21 functions not only in classical cell cycle control but also plays important roles in immune cell activation and regulation. Therefore, we examined p21 protein levels by Western Blot. The results showed that CXCL8 stimulation significantly promoted p21 protein expression in neutrophils, an effect inhibited by the CXCR1/2 antagonist Reparixin, and the p21 inhibitor UC2288 attenuated CXCL8-induced Cit-H3 expression in neutrophils, which confirmed that p21 functions downstream of the CXCL8-CXCR1/2 signaling pathway. To further define the hierarchical relationship within this pathway, we employed the NADPH oxidase inhibitor DPI to block ROS production. DPI pretreatment significantly attenuated CXCL8-induced p21 upregulation and Cit-H3 expression, indicating that ROS functions upstream of p21 in the NETosis pathway. These findings, together with the observation that the p21 inhibitor UC2288 blocks NETosis, support a model in which CXCL8-CXCR1/2 signaling triggers ROS production, which in turn upregulates p21 and promotes NET formation. This mechanistic model is strongly supported by clinical evidence from the TCGA–CESC cohort, in which CXCL8 expression was not only associated with poor prognosis but also showed a significant positive correlation with neutrophil infiltration. Drug sensitivity analysis further suggested that patients with low CXCL8 expression exhibited higher sensitivity to multiple chemotherapeutic agents and immunosuppressants. This observation raises the intriguing possibility that CXCL8-driven NETosis may contribute to therapeutic resistance by forming a physical barrier that limits drug penetration or by establishing an immunosuppressive microenvironment that attenuates the activity of immune-based therapies. Collectively, these clinical correlations not only validate our mechanistic findings but also position the CXCL8–NETs axis as a dual target, whose inhibition could both restrain tumor progression and sensitize tumors to existing therapies. An apparent paradox warrants further discussion. Despite prominent lymphocyte infiltration in the tumor microenvironment of high-risk HPV-driven cervical cancer, a high Treg/CD8^+^ T cell ratio is observed. The infiltrated lymphocytes are largely functionally exhausted and fail to eliminate tumor cells effectively. In contrast, prominent neutrophilic infiltration and NETosis are frequently linked to more aggressive, immunosuppressive phenotypes. Neutrophils are key effectors of the innate immune system, and NETosis represents one of their fundamental defense mechanisms against pathogens (39). When neutrophils encounter stimulatory signals—whether microbial components, inflammatory cytokines, or tumor-derived factors—they become activated and may undergo NETosis as part of the innate immune response (40). How, then, does HPV infection correlate with enhanced NETosis? We propose that these observations are not mutually exclusive. Persistent HPV infection drives chronic inflammation, which recruits diverse immune cells including both T cells and neutrophils. In this context, HPV-transformed epithelial cells secrete chemokines such as CXCL8, which preferentially recruit and activate neutrophils. Thus, the NETosis observed in HPV-positive tumors may represent a component of the HPV-driven inflammatory response, reflecting the activation of innate immunity in the tumor microenvironment, rather than a direct effect of viral proteins on neutrophils. Our *in vitro* data support this interpretation: HPV-positive CC cells secrete high levels of CXCL8, which acts on neutrophils through CXCR1/2 to induce NETosis, while viral proteins themselves were not directly tested. We acknowledge that our data cannot fully distinguish between direct and indirect effects of HPV, and this represents a limitation of the present study. While our findings support a tumor-promoting function of NETs in cervical cancer, NETs have been reported to exert antitumor effects in other tumor types. For example, in melanoma, *in vitro* assays have shown that melanoma cells attach to NETs via integrin-mediated adhesion, and that NETs inhibit tumor cell migration (41). Moreover, co-culturing of NETs with melanoma cells exerted a cytotoxic effect, leading to necrosis. These observations indicate that the biological impact of NETs may vary across tumor types and microenvironments. In addition, NETs have been reported to facilitate angiogenesis and suppress T-cell responses in certain tumor types, yet under other circumstances they can also enhance antigen presentation and stimulate innate immunity. This functional heterogeneity further underscores that the net outcome of NETosis is highly dependent on the local microenvironment. Thus, the net effect of NETosis likely depends on the specific tumor context, including the origin of tumor cells, the composition of the immune infiltrate, and the local inflammatory milieu.

A caveat is that our *in vivo* models do not fully recapitulate HPV-positive cervical cancer. U14 cells are HPV-negative, and NETs were induced by PMA or LPS rather than tumor-derived signals. Thus, these *in vivo* experiments mainly show that NETs can promote tumor growth and lung colonization in general, while our *in vitro* data demonstrate that HPV-positive cells drive NETosis through CXCL8-CXCR1/2-ROS-p21. Taken together, the *in vivo* and *in vitro* findings suggest that this axis may contribute to cervical cancer progression. In addition, the detectable levels of serum MPO-DNA complexes in CC patients raise the possibility that circulating NETs may serve as a candidate biomarker for further evaluation, although this requires validation in larger independent cohorts (**Figure 6P**). Clinically, targeting this axis—by degrading existing NETs with DNase 1 or by blocking their formation with the CXCR1/2 inhibitor Reparixin—may represent a valuable adjunct strategy, while the selective elevation of serum MPO–DNA complexes in HPV-positive patients offers a foundation for developing circulating NETs as novel biomarkers. One caveat deserves an emphasis: tumor-associated macrophages can also generate macrophage extracellular traps (METs) (42, 43), whose DNA scaffold, histones and granular enzymes overlap substantially with those of NETs (44). This compositional and functional convergence raises the possibility that NETs and METs form a cooperative trap network within the tumor microenvironment, synergistically amplifying tumor-promoting signals. Several limitations are still in the present study. Our *in vivo* data support a general role for NETs in promoting tumor growth and lung colonization, but do not constitute direct validation of the HPV-CXCL8-ROS-p21-NETs axis which is validated *in vitro*. Nevertheless, our *in vivo* and *in vitro* findings together provide complementary support for the involvement of this axis in cervical cancer.

## 5 Conclusion

This study systematically elucidates the critical role of NETs in the progression of HPV-positive CC, revealing the pathogenic axis of “HPV-CXCL8-CXCR1/2-ROS-p21-NETs”. NETs were found to promote tumor proliferation and angiogenesis, and to facilitate experimental lung colonization in a tail-vein model. Their levels were also correlated with adverse clinicopathological features. These findings not only deepen the understanding of the pathogenesis of HPV-associated CC but also provide an important theoretical basis for developing novel NETs-targeted therapeutic strategies and prognostic biomarkers. Our *in vitro* data suggest that HPV-positive CC cells may promote NETosis through this pathway, while *in vivo* data indicate that NETs can facilitate tumor growth and lung colonization. However, because our single-cell sequencing analysis was based on a small number of samples and we used surrogate cell models (such as HL-60 cells and mouse U14 tumors) rather than human patient-derived materials, these results should be viewed as preliminary. They should be considered a starting point, and will need confirmation in better models and larger patient cohorts.

## Data Availability

The datasets presented in this study can be found in online repositories. The names of the repository/repositories and accession number(s) can be found in the article/**Supplementary Material**.

## Supplementary material

Supplementary Figure 1 Expression of CD66b, MPO in HPV-positive cervical cancer and HPV-negative cervical cancer tissues was detected by IHC. The left panel displays a representative area at 200× magnification; the right panel depicts the same field at 400× magnification.

Supplementary Figure 2 **(A)** Giemsa staining of HL-60 cells treated with 1 μmol/L ATRA to assess neutrophil differentiation at days 1, 3, 5, and 7. The inset shows a representative area at 200× magnification; the main panel shows a view at 400× magnification. **(B)** Western blot analysis of Cit-H3 in neutrophils treated with or without PMA and DNase 1 for 4 hours. β-Actin was used as a loading control. **(C)** Body weight changes of mice in each group of the subcutaneous xenograft tumor model. **(D)** H&E staining of lymph nodes from mice in the Neu+U14, Neu (PMA)+U14, and Neu (PMA+DNase 1) +U14 groups (left panel: 40× magnification; right panel: 200× magnification). **(E)** Body weight changes of mice in each group of the tail vein metastasis tumor model. **(F)** H&E staining of liver tissues from mice in the U14, LPS+U14, and LPS+U14+DNase 1 groups in the tail vein metastasis model (left panel: 40× magnification; right panel: 200× magnification). Data are presented as mean ± SD. **p* < 0.05, ***P* < 0.01, ****P* < 0.001.

Supplementary Figure 3 **(A)** Overall survival curves of cervical cancer patients stratified by high versus low CXCL8 expression (median cutoff). **(B)** Box plots of immune cell infiltration abundance between CXCL8 high- and low-expression groups, estimated by the CIBERSORT algorithm. **(C)** Correlation between CXCL8 expression and the abundance of immune cells. **(D)** Box plots of drug sensitivity (IC_5__0_) between CXCL8 high- and low-expression groups based on the GDSC database. **p* < 0.05, ***P* < 0.01, ****P* < 0.001.
